# Supplementary material for: Rapid turnover of life-cycle-related genes in the brown algae
Source: Genome Biol. 2019 Feb 14;20:35. doi: 10.1186/s13059-019-1630-6 (PMC6374913; doi:10.1186/s13059-019-1630-6)
Supplement: Supplementary file 3 — Protein alignments, corresponding Gblocks html file and CDS sequences for the lineage conserved gametophyte-biased genes. (ZIP 2109 kb) [file 13059_2019_1630_MOESM3_ESM.zip › Con_GA_alignments/Ec-14_003630.aa_ali.fasta-gb.htm]

Ec-14\_003630.aa.fasta.trans.aa\_ali.fasta


## Gblocks 0.91b Results

Processed file: **Ec-14\_003630.aa.fasta.trans.aa\_ali.fasta**  
Number of sequences: **2**  
Alignment assumed to be: **Protein**  
New number of positions: **408** (selected positions are underlined in blue)

```
                         10        20        30        40        50        60
                 =========+=========+=========+=========+=========+=========+
Ec-14_003630     ------------------------------------------------------------
MSTRG.27131.1    ACLLVIAGDASGGDDETCSAEECVDIEVYQPPYDFLEVTSTNIGNAITVRFNLASRDAYS
                                                                             


                         70        80        90       100       110       120
                 =========+=========+=========+=========+=========+=========+
Ec-14_003630     ----------------------------MNFRIGCWFYFGRTVELGQTFVCSSDPDPQLS
MSTRG.27131.1    GDWGWVLGGVDASLTVEEANRLSADEANDAYDERCWFYFGRAVELEQIFLCSSEPDPWLS
                                                   ##########################


                        130       140       150       160       170       180
                 =========+=========+=========+=========+=========+=========+
Ec-14_003630     AFQNTPTLRPYTYGRFEMYLSTAGNGSLWFGISEQYKYAMVTNFYTCGVTVQPLDSTTVT
MSTRG.27131.1    SYQNTPTLRAYPYGWFQIYLSAAGNESLWFGEHELYTYTMVTHFYTCGVTVQPLNATTVT
                 ############################################################


                        190       200       210       220       230       240
                 =========+=========+=========+=========+=========+=========+
Ec-14_003630     TTSDVLITAIPEQGELCDLGDGIDLQVRSDEFSANIEDVGAWIYFDGRSLNVSHEETRLS
MSTRG.27131.1    TTSDIVVTAVAEQGDHCDVGDGIELSIYSGEVSANIEDVGAWIYFTGRNVNVSREETSLS
                 ############################################################


                        250       260       270       280       290       300
                 =========+=========+=========+=========+=========+=========+
Ec-14_003630     LRFAYTGEHFINVYLASGARLASMNITVVAAAPYAPACEVTGRPPSSAPEGSSVVASLTA
MSTRG.27131.1    IRLAYKGDHVILVSLASGAMLAKMNVTVVAADPYAPACEVTERPPSSSPEGSSVLVSFRA
                 ############################################################


                        310       320       330       340       350       360
                 =========+=========+=========+=========+=========+=========+
Ec-14_003630     RDFYGNSI-TEQWGDNAFQAWTYAGGVGDNVSAATVHDTGNGSYRVVSDTLGDAA-TTFL
MSTRG.27131.1    RDFYDNWISSESSGGDSFRAWSYAT-AANTTASANVFDLGNGSFTVVSDALDEAGGTTFL
                 ########  ##############         #####################  ####


                        370       380       390       400       410       420
                 =========+=========+=========+=========+=========+=========+
Ec-14_003630     FVERDALGVPGSPFEVYVFGPANCAVMSTVGDCQSDLWRPVSQQWAARSPCIGQFDDPEH
MSTRG.27131.1    FVERDALGIPGSPFEVYVFGPASCDVVSTVGDCQPDLRRMVSQEWAANSPCLGQFDDPEH
                 ############################################################


                        430       440       450       460       470       480
                 =========+=========+=========+=========+=========+=========+
Ec-14_003630     SGIGCGYIPLKSDLGTTIVVCAAFAGLYAAVILWWIRRERWAVIVKLSQPFVCQLFLAGC
MSTRG.27131.1    TSVACGSVPVGSGLGTTMVVTAAFAGLYAAVFLRWIRRNRRTVIVKLSQPFVCQLFLAGC
                 ############################################################


                        490       500       510       520       530       540
                 =========+=========+=========+=========+=========+=========+
Ec-14_003630     ILLNITAAFLVGVASDPSCVMQPWMLHIGLTFTSG-------------------------
MSTRG.27131.1    ILLNITASLHVEMAMHAACVVRPWMMHIGLTFTSGCLYMKTYRVHRIFNNSSKLSRVSIT
                 ###################################                         


                        550       560       570       580       590       600
                 =========+=========+=========+=========+=========+=========+
Ec-14_003630     ------------------------------------------------------------
MSTRG.27131.1    DRQLVSWVMKLVAVDVAFLIIWTMVDRPRRVDVVEFIGDVEVVNSECMERSDIPIFRSIL
                                                                             


                        610       620       630       640       650       660
                 =========+=========+=========+=========+=========+=========+
Ec-14_003630     ------------------------------------------------------------
MSTRG.27131.1    WSWKACLNCFGVVMAAKTWHCEDGVGESQPICLCVYNTTLLGLVNLALQVFLSGNSNTVV
                                                                             


                        670
                 =========+
Ec-14_003630     ----------
MSTRG.27131.1    GIGASAFTLX
```

```
Parameters used
Minimum Number Of Sequences For A Conserved Position: 2
Minimum Number Of Sequences For A Flanking Position: 2
Maximum Number Of Contiguous Nonconserved Positions: 8
Minimum Length Of A Block: 10
Allowed Gap Positions: None
Use Similarity Matrices: Yes
```

```
Flank positions of the 4 selected block(s)
Flanks: [95  308]  [311  324]  [334  354]  [357  515]  

New number of positions in Ec-14_003630.aa.fasta.trans.aa_ali.fasta-gb:  408  (60% of the original 670 positions)
```
